# Effect of continuous positive airway pressure on symptoms of anxiety and depression in patients with obstructive sleep apnea

**DOI:** 10.1007/s11325-020-02234-7

**Published:** 2020-10-24

**Authors:** Ragnhild Stokke Lundetræ, Ingvild West Saxvig, Sverre Lehmann, Bjørn Bjorvatn

**Affiliations:** 1 Department of Global Public Health and Primary Care, University of Bergen, Kalfarveien 31, N-5018 Bergen, Norway; 2 Centre for Sleep Medicine, Haukeland University Hospital, Bergen, Norway; 3 Norwegian Competence Center for Sleep Disorders, Haukeland University Hospital, Bergen, Norway; 4 Department of Clinical Science, University of Bergen, Bergen, Norway

**Keywords:** Anxiety, CPAP, Depression, HADS, OSA, Sleep apnea

## Abstract

**Purpose:**

The objective was to assess the effect of continuous positive airway pressure (CPAP) on symptoms of anxiety and depression in patients with obstructive sleep apnea (OSA). We hypothesized a decrease in symptoms at follow-up, but that improvement relied on CPAP adherence.

**Methods:**

The sample comprised 468 patients (mean age 55.5 years (SD = 12.0), 72% men) with OSA who received CPAP at a Norwegian hospital. OSA was diagnosed according to standard respiratory polygraphy. Mean baseline respiratory event index (REI) was 28.4 (SD = 20.6). Symptoms of anxiety and depression were assessed prior to CPAP treatment and at follow-up after a median of 20 weeks, range 6–52 weeks, with the Hospital Anxiety and Depression Scale (HADS). Patients were classified as CPAP adherent (≥ 4 h per night) or non-adherent (< 4 h per night).

**Results:**

There was a significant decrease in anxiety scores from baseline (mean = 5.16, SD = 3.94) to follow-up (mean = 4.76, SD = 3.81), *p* < 0.001. Similarly, depression scores decreased from baseline (mean = 4.31, SD = 3.66) to follow-up (mean = 3.89, SD = 3.69), *p* < 0.001. Cohen’s d (0.19 and 0.18, respectively) indicated small effect sizes. The reduction in anxiety scores did not depend on CPAP adherence (no interaction effect *F*(1, 466) = 0.422, *p* = 0.516), whereas the reduction in depression scores were seen only in the CPAP adherent group (interaction effect *F*(1, 466) = 7.738, *p* = 0.006).

**Conclusions:**

We found a decrease in symptoms of anxiety and depression from baseline to follow-up of CPAP treatment. The improvement in symptoms of depression was depending on CPAP adherence. This underlines the importance of adherence for optimal effect of CPAP treatment.

## Introduction

Obstructive sleep apnea (OSA) is a common sleep disorder characterized by breathing pauses during sleep [1]. Typical symptoms are snoring and excessive daytime sleepiness [2]. OSA can have severe health implications such as increased risk of diabetes, obesity, cardiovascular diseases, and premature death [3–5]. OSA is also associated with increased levels of psychiatric comorbidities, such as anxiety and depression [1, 6]. Anxiety and depression are widespread public health challenges and are together with substance use disorders leading causes of years lived with disability worldwide [7]. In a review including 55 publications from 1995 to 2006 looking at anxiety and depression in OSA patients, the prevalence of anxiety varied from 11 to 70% and the prevalence of depression varied from 7 to 63% [6]. The large range in prevalence was related to many factors, including the use of different mood scales. Furthermore, the review addressed several limitations of the included studies, such as low number of participants, validity issues with the mood scales, and different methods for diagnosing OSA.

Continuous positive airway pressure (CPAP) is the first-line treatment for OSA, and is indicated for patients with moderate and severe OSA or mild OSA with comorbidities (cardiovascular diseases, cognitive dysfunction, mood disorders, etc.) [8]. CPAP is extremely effective in keeping the airways open, and thereby reducing the apneas. It has been shown to reduce cardiovascular risk and is proven to be cost-effective in patients with moderate and severe OSA [9, 10], whereas the effect on anxiety and depression remains uncertain. A previous study on the impact of OSA treatment on depression shows that depression scores in patients with comorbid OSA and depression improve with CPAP [11]. Similarly, the review by Saunamäki and Jehkonen reported that CPAP might improve mood [6]. However, these findings were inconsistent and the authors called for long-term follow-up studies to verify the effectiveness. A meta-analysis implies that the improved scores in anxiety and depression may be mediated by patients’ expectations and contact with healthcare providers [12]. A recent systematic review concludes that the CPAP treatment has good effect on depression, but not anxiety [13]. However, the study included patients with moderate or severe OSA and coexisting cardiovascular disease only, thus, the results may not be generalizable to the overall OSA population. Hence, there is need for prospective studies in large OSA populations to further elucidate how psychiatric comorbidities relate to OSA, and whether treating OSA reduces the risk and severity of such disorders.

Against this backdrop, the primary objective of the present study was to assess the effect of CPAP on symptoms of anxiety and depression in a large group of patients with OSA using the validated Hospital Anxiety and Depression Scale (HADS) [14, 15]. We hypothesized a decrease in HADS scores at follow-up after about 3 months with CPAP, but that this improvement depended on CPAP adherence.

## Material and methods

The sample comprised 468 patients diagnosed with OSA and receiving CPAP treatment (Airsense^TM^ 10 Autoset^TM^ or S9 AutoSet^TM^, ResMed Norway AS) at a Norwegian university hospital between 2011 and 2017. In total, 338 (72%) of the patients were men and 130 (28%) were women. Mean age was 55.5 ± 12.0 years (range 21–87). OSA diagnosis was based on standard out-of-center respiratory polygraphy using a type 3 portable monitor (Embletta^TM^ or NOX T3, Resmed Norway AS) as described in previous publications [16]. Scoring rules were in accordance with the 2007 American Academy of Sleep Medicine Manual [17], defining apneas as a reduction of 90% or more of baseline nasal airflow with a duration of at least 10 s. Hypopneas were defined as a nasal flow reduction of 30–90% of baseline, lasting at least 10 s accompanied by an oxygen desaturation of ≥ 4%. Obstructive sleep apnea was classified in accordance with the respiratory event index (REI), which is the number of apneas and hypopneas per hour of monitoring, i.e., no OSA (REI < 5), mild OSA (REI 5–14.9), moderate OSA (REI 15–29.9), severe OSA (REI ≥ 30). In addition, for mild OSA, presence of symptoms or a relevant comorbidity, such as hypertension or a mood disorder, had to be present. Mean baseline REI in the current sample was 28.4 ± 20.6 (range 5.0–135.2).

Prior to CPAP treatment, the patients completed a questionnaire including questions about age, sex, marital status, mental health, and whether they had been diagnosed with hypertension, diabetes mellitus, myocardial infarction, stroke, chronic obstructive pulmonary disease, or angina pectoris. The same questionnaire was completed at follow-up, which was scheduled after about 3 months. To assess current symptoms of anxiety and depression, the patients completed the validated HADS [15]. The scale consists of 14 separate statements, divided into two seven-item subscales, one for anxiety (HADS-A) and one for depression (HADS-D), both reflecting non-vegetative symptoms during the last week. Each statement has four response alternatives, ranging from a healthy state (0) to maximum symptom severity (3). The composite HADS score for either anxiety or depression is consequently ranging from 0 to 21. A score of 8 or higher on each subscale indicates that the patient may suffer from anxiety or depression, respectively [14, 15]. At follow-up, patients were categorized as CPAP adherent or non-adherent, based on their average CPAP use the last 90 nights as measured by the CPAP device. Adherence was defined as CPAP use ≥ 4 h per night [13, 18, 19].

The study was approved by The Regional Committee for Medical and Health Research Ethics, Health Region West (REC 2018/337). Written informed consent was obtained from all patients.

### Statistics

The IBM SPSS Statistics, version 25.0 was used for the data analyses. Follow-up scores for HADS were missing for 18.6% (anxiety) and 16.2% (depression) of the cases. These missing values were replaced using the conservative last observation carried forward method [20].

Paired-samples *t* tests were conducted to evaluate the impact of CPAP on the anxiety (HADS-A) and depression (HADS-D) scores, respectively. In addition, separate analyses were conducted including only patients with HADS-A ≥ 8 (possible anxiety) and HADS-D ≥ 8 (possible depression) at baseline. Normality checks were carried out and the assumptions met. Furthermore, mixed between-within subjects ANOVAs (group × time) were conducted in order to explore the impact of CPAP adherence (adherence vs non-adherence) and REI severity (REI ≥ 30 vs REI < 30), respectively, on HADS scores (baseline vs follow-up). Interaction effects were further explored using the pairwise *t* tests for simple effects. Cohen’s *d* for paired values was calculated as a measure of effect size (*d* = M_1_−M_2_/SD pooled), considering *d* = 0.2 as small, *d* = 0.5 as moderate, and *d* = 0.8 as large effect sizes [21]. Mixed between-within ANOVAs were also conducted in order to explore the impact of follow-up time (< 20 weeks vs ≥ 20 weeks) on HADS scores (baseline vs follow-up). We used median split to dichotomize the follow-up variable. Furthermore, mixed between-within ANOVAs were conducted in order to explore the impact of the comorbid diseases (e.g., hypertension vs no hypertension) on HADS scores (baseline vs follow-up). Significance level was set to 0.05 for all analyses.

## Results

Descriptive statistics at baseline are presented in Table 1. Although follow-up was scheduled at around 3 months, the actual time for follow-up ranged from 6.0 to 51.9 weeks (mean 23.7 weeks, median 20.0 weeks). This was mostly due to patients re-scheduling their appointment. Mean CPAP adherence at follow-up was 4:20 h use per night (range 0:00 to 9:40 h, SD = 2:24).

**Table 1:** Descriptive statistics at baseline among Norwegian patients with obstructive sleep apnea

Number of patients	468
Sex, males	71.2% (*n* = 338)
Age (mean ± SD)	55.5 ± 12.0
REI ( mean ± SD)	28.4 ± 20.6
REI ≥ 30	35.7% (*n* = 167)
HADS-A (mean ± SD)	5.2 ± 4.0
HADS-D (mean ± SD)	4.3 ± 3.7
HADS-A ≥ 8	26.3% (*n* = 123)
HADS-D ≥ 8	17.5% (*n* = 82)
Hypertension^1^	49.9% (*n* = 229)
Diabetes mellitus^1^	15.1% (*n* = 69)
Myocardial infarction^1^	7.9% (*n* = 36)
Stroke^1^	3.4% (*n* = 15)
COPD^1^	5.1% (*n* = 23)
Angina pectoris^1^	5.5% (*n* = 24)

*SD*, standard deviation; *REI*, respiratory event index; *HADS-A*, Hospital Anxiety and Depression Scale-Anxiety subscale (0–21 scale); *HADS-D*, Hospital Anxiety and Depression Scale-Depression subscale (0–21 scale); *COPD*, chronic obstructive pulmonary disease

^1^Based on self-report

Overall, there was a statistically significant decrease in both HADS-A and HADS-D scores from baseline to follow-up, *p* < 0.001 (Table 2). Cohen’s *d* for repeated measures (0.19 for anxiety and 0.18 for depression) indicated small effect sizes. When the patients with HADS-A ≥ 8 and HADS-D ≥ 8 were analyzed separately, both groups had a statistically significant decrease in HADS scores from baseline to follow-up, with moderate effect sizes (Cohen’s *d* = 0.50 for anxiety and 0.61 for depression) (Table 2).

**Table 2:** Comparison of HADS-A and HADS-D scores at baseline and follow-up among 468 Norwegian patients treated with CPAP for obstructive sleep apnea

	Baseline	Follow-up	Mean difference	Effect size	*t* test
	Mean ± SD	Mean ± SD	Mean (95% CI)	Cohen’s *d*	*p*
HADS-A, all patients (*n* = 468)	5.16 ± 3.94	4.76 ± 3.81	0.40 (0.21–0.58)	0.19	< 0.001
HADS-D, all patients (*n* = 468)	4.31 ± 3.66	3.89 ± 3.69	0.42 (0.20–0.63)	0.18	< 0.001
HADS-A ≥ 8 (*n* = 123, 26.3%)	10.50 ± 2.50	9.22 ± 3.32	1.28 (0.82–1.73)	0.50	< 0.001
HADS-D ≥ 8 (*n* = 82, 17.5%)	10.64 ± 2.27	8.84 ± 3.64	1.80 (1.16–2.45)	0.61	< 0.001

*HADS-A*, Hospital Anxiety and Depression Scale-Anxiety subscale (0–21 scale); *HADS-D*, Hospital Anxiety and Depression Scale-Depression subscale (0–21 scale); *CPAP*, continuous positive airway pressure; *SD*, standard deviation; *CI*, confidence interval

Table 3 shows the results from the mixed between-within subjects ANOVAs conducted to assess the impact of CPAP adherence on the changes in HADS scores. There was no significant interaction effects for HADS-A, *F*(1, 466) = 0.422, *p* = 0.516. There was a main effect for time, with both CPAP adherence groups showing a reduction in HADS-A (anxiety) scores from baseline to follow-up (Table 3). In contrast, for symptoms of depression, there was a significant interaction effect, *F*(1, 466) = 7.738, *p* = 0.006, indicating that the reduction in HADS-D scores was larger in the adherent group than in the non-adherent group (Table 3). Subsequent analyses of simple effects revealed a significant reduction in HADS-D scores for the adherent group (*p* < 0.001, Cohen’s *d* = 0.31), whereas the non-adherent group had no reduction in HADS-D scores (*p* = 0.823, Cohen’s *d* = 0.02). There was a significant main effect of group in both HADS-A and HADS-D, with higher HADS scores in the non-adherent group than in the adherent group (*p* = 0.011 and 0.004, respectively).

**Table 3:** Comparison of HADS-A and HADS-D scores at baseline and follow-up between and within adherence groups among 468 Norwegian patients treated with CPAP for obstructive sleep apnea

	Baseline	Follow-up	Mean difference	Effect size	*t* test	ANOVA (*p* value) (group × time)		
	Mean ± SD	Mean ± SD	Mean (95% CI)	Cohen’s *d*	*p*	Time	Group	Interaction
HADS-A								
Adherent^a^ (*n* = 283)	4.83 ± 3.85	4.38 ± 3.72	0.45 (0.20–0.69)	0.21	*< 0.001*	*< 0.001*	*0.011*	0.516
Non-adherent^b^ (*n* = 185)	5.66 ± 4.03	5.35 ± 3.89	0.31 (0.04–0.60)	0.16	*0.028*			
HADS-D								
Adherent (*n* = 283)	4.06 ± 3.57	3.40 ± 3.50	0.66 (0.41–0.91)	0.31	*< 0.001*	*0.002*	*0.004*	*0.006*
Non-adherent (*n* = 185)	4.69 ± 3.77	4.65 ± 3.86	0.04 (− 0.34–0.42)	0.02	0.823			

^a^Adherent was defined as using the CPAP ≥ 4 h per night

^b^Non-adherent was defined as using the CPAP < 4 h per night

*HADS-A*, Hospital Anxiety and Depression Scale-Anxiety subscale; *HADS-D*, Hospital Anxiety and Depression Scale-Depression subscale; *CPAP*, continuous positive airway pressure; *SD*, standard deviation; *CI*, confidence interval

Significant values are shown in italics

Table 4 shows the results from the mixed between-within subjects ANOVAs conducted to assess the impact of REI severity on changes in HADS scores. There was no significant group × time interaction effect for HADS-A, *F*(1, 466) = 2.408, *p* = 0.121. Similarly, there was no significant interaction between REI groups and time for HADS-D, *F*(1, 466) = 0.118, *p* = 0.732. There was a main effect for time, with both REI groups showing a reduction in HADS-D scores from baseline to follow-up (Table 4). Furthermore, there was a significant main effect of group in both HADS-A and HADS-D, with higher HADS scores in the group with REI ≥ 30 than in the group with REI < 30 (*p* = 0.014 and 0.027, respectively).

**Table 4:** Comparison of HADS-A and HADS-D scores at baseline and follow-up between and within REI severity groups among 468 Norwegian patients treated with CPAP for obstructive sleep apnea

	Baseline	Follow-up	Mean difference	Effect size	*t* test	ANOVA (*p* value) (group × time)		
	Mean ± SD	Mean ± SD	Mean (95% CI)	Cohen’s *d*	*p*	Time	Group	Interaction
HADS-A								
REI ≥ 30^a^ (*n* = 167)	4.49 ± 3.85	4.29 ± 3.79	0.20 (− 0.08–0.48)	0.11	0.166	*< 0.001*	*0.014*	0.121
REI < 30^b^ (*n* = 301)	5.53 ± 3.95	5.02 ± 3.81	0.51 (0.26–0.75)	0.23	*< 0.001*			
HADS-D								
REI ≥ 30 (*n* = 167)	3.86 ± 3.33	3.39 ± 3.49	0.47 (0.13–0.81)	0.21	*0.008*	*< 0.001*	*0.027*	0.732
REI < 30 (*n* = 301)	4.56 ± 3.81	4.17 ± 3.78	0.39 (0.11–0.67)	0.16	*0.006*			

*REI*, respiratory event index; *HADS-A*, Hospital Anxiety and Depression Scale-Anxiety subscale; *HADS-D*, Hospital Anxiety and Depression Scale-Depression subscale; *CPAP*, continuous positive airway pressure; *SD*, standard deviation; *CI*, confidence interval

^a^REI ≥ 30 is defined as severe obstructive sleep apnea

^b^REI < 30 is defined as mild to moderate obstructive sleep apnea

Significant values are shown in italics

In terms of follow-up time (< 20 weeks vs ≥ 20 weeks), there was no significant group × time interaction effects for HADS-A, *F*(1,466) = 0.080, *p* = 0.778. Similarly, there was no significant group × time interaction effects for HADS-D, *F*(1,466) = 3.491, *p* = 0.062. Furthermore, there was no significant group × time interaction effects for neither HADS-A nor HADS-D in terms of being diagnosed with hypertension, *F*(1,457) = 0.318, *p* = 0.573 and *F*(1,457) = 0.053, *p* = 0.819, respectively. Similarly, no such interaction effects were found for any of the other comorbid diseases (diabetes mellitus, myocardial infarction, stroke, COPD, or angina pectoris).

## Discussion

We found a significant decrease in symptoms of anxiety and depression at follow-up compared to baseline. Thus, our findings are in agreement with our initial hypothesis of a reduction in both HADS-A and HADS-D scores at follow-up after CPAP treatment. The second hypothesis was that the improvement would be dependent on CPAP adherence. This was the case for depression, but not for anxiety.

Previous studies investigating the effect of CPAP on symptoms of anxiety and depression in patients with OSA have been inconclusive. Some [11, 12] but not all [22] report that treatment with CPAP improves symptoms of anxiety and depression. A review reported that depressive symptoms were reduced in four out of seven studies and symptoms of anxiety in two out of four studies [6]. However, low number of participants, validity issues with the different mood scales, and different methods for diagnosing OSA were addressed as limitations in these studies. A recent study reported no change in depression symptoms measured with Beck Depression Inventory-Fast Screen (BDI-FS) after 3 months of CPAP use [23]. When measuring with State-Trait Depression Inventory (IDER), however, the researchers found a statistically significant decrease in depression symptoms. Furthermore, they reported a significant reduction in anxiety-trait symptoms measured with State-Trait Anxiety Inventory (STAI) [23]. A strength of this study was using scales adjusted to OSA patients, including only non-overlapping symptoms of anxiety and depression. However, the sample comprised only 48 patients, and there were no present anxiety or depression symptoms of clinical relevance at baseline, thus, a potential decrease in these symptoms may not have been properly assessed. The current findings among 468 patients with OSA support the notion about reduced HADS scores after CPAP.

Although the overall HADS scores decreased for both symptoms of anxiety and depression, the improvement was only depending on CPAP adherence for depression. This finding is in agreement with Zheng et al., which concluded that CPAP provides a good treatment for depression, but not for anxiety [13]. The reason for this is unclear, but pathophysiological mechanisms such as apnea-induced sleep fragmentation, excessive daytime sleepiness, and decreased gray matter due to hypoxemia may be reversed by CPAP treatment [24, 25], whereas the CPAP may have an anxiety-inducing effect from the mask application [13].

There was a significant group difference between adherent and non-adherent patients in terms of higher HADS scores in the non-adherent group. This is in accordance with previous studies finding lower adherence to CPAP in patients with higher HADS scores, both for anxiety and depression [26]. Anxiety and depression may leave the patients unmotivated or less tolerant to the CPAP therapy, and further studies should investigate whether treatment of psychiatric comorbidities may enhance adherence to OSA treatment.

As expected, the effect size was larger when patients with possible anxiety (HADS-A ≥ 8) and depression (HADS-D ≥ 8) were analyzed separately. As HADS scores at baseline for the whole group were low for both anxiety and depression, many patients had a limited potential for further reductions. Our study suggests that patients with high HADS scores at baseline will benefit more from CPAP on their psychiatric comorbidity. However, this effect could also be due to “regression to the mean” [27], meaning that patients with high HADS scores at baseline are more likely to get closer to the mean if measured a second time, independently of CPAP adherence.

In a cross-sectional study among OSA patients, Bjorvatn et al. found that symptoms of anxiety and depression were negatively associated with OSA severity, after adjusting for relevant confounders such as sex, age, smoking, alcohol consumption, and obesity [28]. In the present study, however, there was no significant interaction between OSA severity and time for either HADS-A or HADS-D. This finding indicates that patients might benefit from CPAP in terms of symptoms of anxiety and depression regardless of OSA severity.

As expected, we found a clearly higher prevalence of possible anxiety (26.3%) and depression (17.5%) among these OSA patients, compared to means of 12.6% for anxiety and 4.4% for depression in the general Norwegian population [29]. The mean HADS-A and HADS-D scores were comparable to another Norwegian study in OSA patients, where HADS mean scores for the whole patient group were 5.4 for anxiety and 4.8 for depression [26].

The psychopathology underlying the association between OSA and mental disorders is unclear. Previous studies showing a positive association between OSA and anxiety/depression are mostly cross-sectional, thus, we cannot tell cause from effect. Furthermore, it is unclear whether depression is a primary consequence of OSA or if it occurs secondary to OSA-related symptoms (e.g., excessive sleepiness, fatigue, sleep problems, irritability) or to other OSA-related factors such as obesity and cardiovascular diseases [6]. Treating OSA with CPAP has proven to be effective on several of these symptoms [10, 22], and findings in the present study provide evidence that CPAP may have a positive impact on symptoms of anxiety and depression. Hence, in clinics treating patients with OSA, follow-up consultations provided by trained healthcare providers are important to ensure CPAP adherence. However, patients with psychiatric comorbidity should get a psychiatric evaluation if the treatment of OSA does not result in mood improvement. For optimal treatment outcomes in patients with OSA and comorbid psychiatric disorders, treatment of both disorders should be considered [30].

Some strengths and limitations of the present study should be noted. The relatively large sample size provided statistical power to the analyses. Another strength was the use of a well-validated questionnaire for anxiety and depression. A recent study highlights the need for studies using valid scales that not include somatic symptoms found in and caused by OSA [23]. In contrast to most other anxiety and depression scales, HADS does not include questions about vegetative symptoms such as sleepiness and fatigue. Sleepiness and fatigue are usually considered symptoms directly related to OSA, thus, a scale focusing on non-vegetative symptoms of anxiety and depression is a strength. As the HADS questionnaire asks about symptoms during the last week, it does not provide formal anxiety or depression diagnoses, only an indication of possible cases. We cannot be sure whether the decrease in HAD scores from baseline to follow-up was due to CPAP treatment or to the therapeutic effect of meeting trained health care personnel. However, the reduction in HADS-D scores was only significant among the patients with CPAP adherence (≥ 4 h/night), which suggests that there was an actual effect of CPAP on symptoms of depression. A limitation was that we lacked valid information on medications for psychiatric conditions which may pose a confounder to the results. Another limitation was that OCST commonly underestimates the number of obstructive respiratory events per hour compared to polysomnography, as polygraphy monitors the number of apneas/hypopneas per hour monitoring rather than total sleep time [1]. This is a diagnostic uncertainty, and we therefore chose to use the term REI instead of apnea-hypopnea index. Furthermore, although follow-up was scheduled at 3 months, the time interval between baseline and follow-up varied considerably. This was mostly due to patients re-scheduling their appointment. We excluded patients attending follow-up in less than 1 month and more than 1 year after baseline, and the statistical analyses showed no significant interaction between follow-up time (< 20 vs ≥ 20 weeks) and decrease in scores for neither HADS-A nor HADS-D. The CPAP device reports an average use over the last 90 nights. However, we do not know how many days of use each patient have, and this was a limitation. Lastly, some patients did not answer all HADS questions at follow-up. This was handled with replacing missing values with scores from baseline (intention-to-treat), which is a conservative procedure that warrants caution when interpreting the data. Such missing substitution may underestimate the effect of CPAP. However, the decrease in symptoms of anxiety and depression remained the same when the patients who did not complete the HADS questionnaire at follow-up were excluded from the analyses. This further supports the conclusions from the present study.

In conclusion, we found a decrease in symptoms of anxiety and depression from baseline to follow-up of CPAP treatment. There was an interaction between CPAP adherence and reduced HADS-D scores from baseline to follow-up, showing that only patients with CPAP adherence had a reduction in symptoms of depression. No such interaction was found for CPAP adherence and HADS-A scores, suggesting that CPAP may not be responsible for the reduction in anxiety symptoms.

## Data Availability

The authors agree all data and statistical information essential to this paper will be made available for review if required.
